# Plasmonic Metasurfaces for Specific SERS Detection of Shiga Toxins

**DOI:** 10.1021/acsami.1c21553

**Published:** 2022-01-19

**Authors:** M. Rippa, D. Sagnelli, A. Vestri, V. Marchesano, B. Munari, D. Carnicelli, E. Varrone, M. Brigotti, R. Tozzoli, M. Montalbano, S. Morabito, J. Zhou, J. Zyss, L. Petti

**Affiliations:** † Institute of Applied Sciences and Intelligent Systems “E. Caianiello” of CNR, 80072 Pozzuoli, Italy; ‡ Dipartimento di Medicina Specialistica, Diagnostica e Sperimentale, Sede di Patologia Generale, Università di Bologna, 40126 Bologna, Italy; § Laboratorio Nazionale di Riferimento per E. coli, Dipartimento di Sicurezza Alimentare, Nutrizione e Sanità Pubblica Veterinaria, Istituto Superiore di Sanità, 00161 Rome, Italy; ∥ Institute of Photonics, Faculty of Science, Ningbo University, 315211 Ningbo, People’s Republic of China; ⊥ Lumière, Matière et Interfaces (LUMIN) Laboratory, Institut d’Alembert, Ecole Normale Supérieure Paris-Saclay, Université Paris Saclay, 91190 Gif sur Yvette, France

**Keywords:** plasmonic, octupolar nanostructure, nanobiosensors, SERS, Shiga toxins, bacterial infections

## Abstract

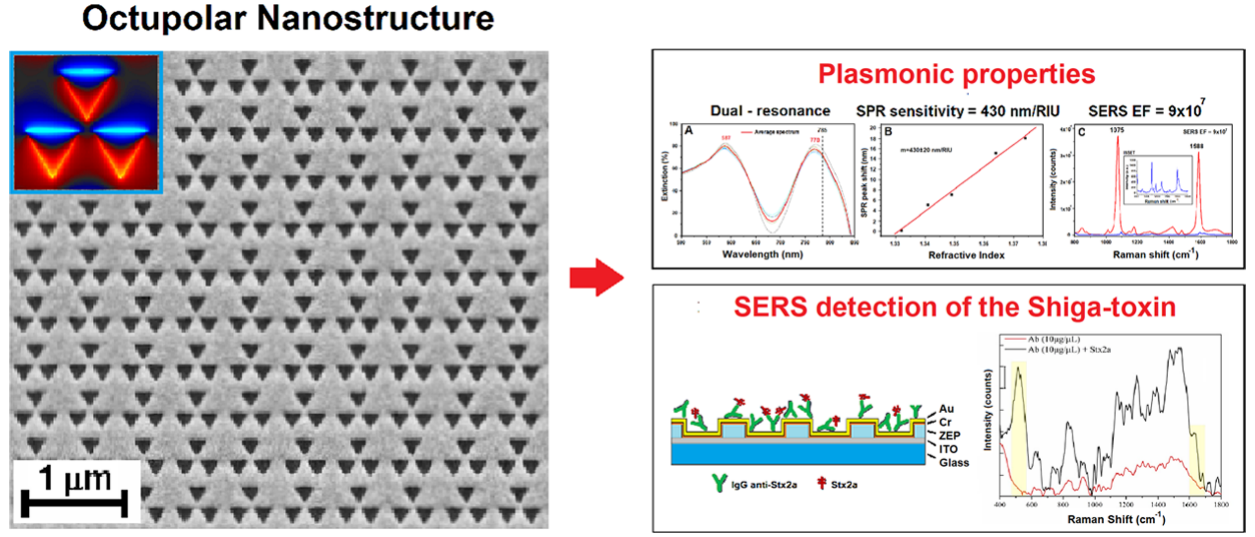

The interest in the development of nanoscale plasmonic technologies has dramatically increased in recent years. The photonic properties of plasmonic nanopatterns can be controlled and tuned via their size, shape, or the arrangement of their constituents. In this work, we propose a 2D hybrid metallic polymeric nanostructure based on the octupolar framework with enhanced sensing property. We analyze its plasmonic features both numerically and experimentally, demonstrating the higher values of their relevant figures of merit: we estimated a surface-enhanced Raman spectroscopy (SERS) enhancement factor of 9 × 10^7^ and a SPR bulk sensitivity of 430 nm/RIU. In addition, our nanostructure exhibits a dual resonance in the visible and near-infrared region, enabling our system toward multispectral plasmonic analysis. Finally, we illustrate our design engineering strategy as enabled by electron beam lithography by the outstanding performance of a SERS-based biosensor that targets the Shiga toxin 2a, a clinically relevant bacterial toxin. To the best of our knowledge, this is the first time that a SERS fingerprint of this toxin has been evidenced.

## Introduction

Plasmonic technologies have attracted intensive research interest in recent years.^1−7^ As a result, plasmon-based devices have achieved significant steps toward real-life applications.^8−12^ The ability to manipulate and control visible and infrared light using nanopatterns based on different geometries of metallic elements has been used to realize, among others, waveguides, coherent light sources, displays, filters, and anti-counterfeiting and sensing systems with unique and outstanding properties.^13,14^ In the case of biological applications, many nanoscale systems based on propagating or localized plasmon resonances have been studied and used to analyze and detect a range of analytes such as bacteria, virus, and molecular systems in general.^15−21^ As widely reported in the literature, the main characteristics of these sensing devices such as sensitivity, limit of detection (LOD), and repeatability depend on their plasmonic properties such as the electromagnetic near-field distribution and the spectral resonances of their nanoelements.^22−27^ These properties can be controlled and tuned by modifying the shape or the size of the unit cell of the nanopattern as well as its geometry. Periodic and aperiodic nanopatterns with unit cells made of both a single nanoelement and a cluster have been widely exploited and used to achieve plasmon-based spectroscopic platforms based on surface-enhanced Raman spectroscopy (SERS), surface plasmon polaritons (SPPs), and localized surface plasmon resonance (LSPR).^28−35^ However, given the crucial role played by geometry, it is getting increasingly important to explore alternative configurations that allow, for example, dual sensing with advanced properties.

At present, one of the best approaches to generate efficient plasmonic surfaces relies on random “roughening” of metal surfaces by colloidal synthesis of nanoparticles resulting in aggregate morphologies. However, the nanosphere size dispersion and position randomness limit the reproducibility of this approach. Electron beam lithography (EBL) is an optimal method for the fabrication of engineered plasmonic substrates. By using EBL, it is possible to fabricate uniform nanopattern substrates by controlling both the shape and position of each particle at the nanoscale. This approach enables the design of engineered nanopattern with ad hoc plasmonic properties and high density of hot spot that can be fabricated with high reproducibility. In addition, the main limitations of the EBL approach, resulting from the lack of cost and time effective schemes for device fabrication and scalability, can be overcome by efficient nanoimprint techniques with nanometer resolutions.

We report here on the spectral and sensing properties of a 2D dual-resonant nanostructure made of a lattice of gold nanocavities (NCs) arranged in a novel octupolar geometry^36^ and fabricated with an EBL technique. We studied its plasmonic features by the use of finite-difference time domain (FDTD)-based simulations. We then demonstrate experimentally the sensing properties of our system. A value of 9 × 10^7^ was evidenced for the SERS enhancement factor (EF), together with a bulk LSPR sensitivity of 430 nm/RIU and a double-plasmonic resonance that make our system extremely useful for spectroscopic investigation in both visible and near-infrared regions. Finally, we tested our nanostructure for a biosensing application of medical interest using it as a SERS substrate to detect the Shiga toxin 2a (Stx2a), a clinically relevant bacterial toxin. The production of Stx2a by pathogenic *Escherichia coli* (Shiga toxin-producing *E. coli*, STEC) is responsible for human infections causing intestinal symptoms, such as watery or bloody diarrhea, and the life-threatening sequela hemolytic uremic syndrome, which represents the major cause of acute renal failure in childhood, with a mortality rate of 3–5%.^37−41^ At present, the diagnosis of STEC infections is directed toward the identification of STEC bacteria in stool samples by polymerase chain reaction amplification of their virulence genes or the detection of circulating antibodies against the surface antigen of a few STEC serogroups. Such approaches are time-consuming and require complex pre-treatment of the sample. Additionally, these methods require that the specimens are sent to a diagnostic center, often a reference laboratory, extending the timeframe from the onset of the disease and its diagnosis, thus delaying the treatment of the infection. Hence, there is a strong need for alternative methods displaying more convenient, rapid, and sensitive features toward toxin detection and identification. In the past years, different kinds of biosensors based on optical and plasmonic methods have been proposed in order to detect dangerous toxins.^42^ Plasmonic systems that exploit the potential of SPR, LSPR, and SERS methods were developed to detect bacterial, fungal, and algal toxins making use of gold films^43^ and gold or silver nanoparticles,^44,45^ their respective nanostructures being achieved by annealing^46^ or evaporation.^47^ However, only few studies have been reported concerning the application of these types of sensors toward the detection of Stx. In 2016, Yamasaki et al.^48^ developed a SPR-based immunosensor biochip using a modified gold thin layer to detect O-antigens belonging to the major STEC serogroups, while, in 2019, Zhang et al.^49^ and, recently in 2020, Wang et al.^50^ used SP-based methods for the detection of Stx.

In this work, using a 2D octupolar nanostructure template, we found, for the first time to the best of our knowledge, the SERS fingerprint of Stx2a and demonstrated the possibility of detecting this toxin in a specific way exploiting an immunosensing strategy. Our method can be a valid alternative to the conventional ones for Stx detection in a real-time label-free analysis and, in addition, can be integrated with other biological on-chip devices to realize portable point-of-care medical diagnostics. Our results suggest that the octupolar pattern investigated is promising to develop rapid, simple, and very sensitive devices for the detection of chemical and biological analytes.

## Results and Discussion

### Octupolar Nanostructure

**Figure 1:**
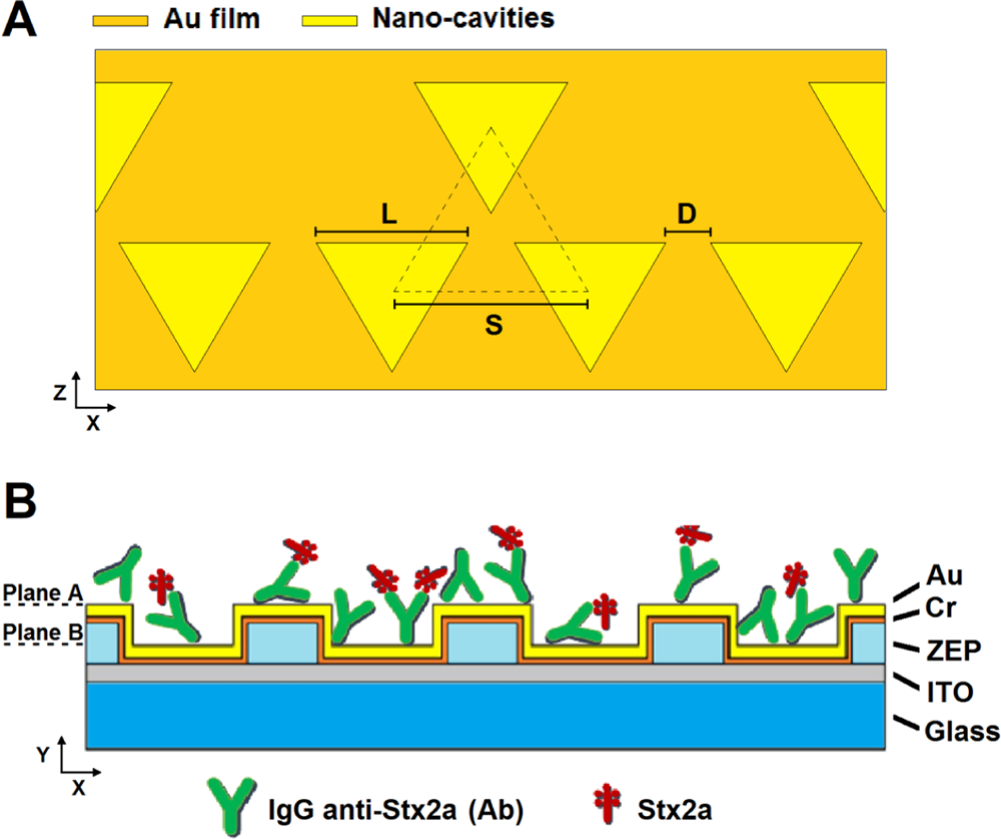
Octupolar nanostructure designed and studied: (A) scheme of the octupolar cell unit (top view) and (B) scheme of the hybrid multilayer (side view) with the link of the complex Ab + Stx2a represented.

The plasmonic properties of nanoscale systems are jointly determined by physicochemical factors, such as their material composition and dielectric properties, and geometric factors, such as symmetry, shape, size, and periodicity. Following a molecular engineering, remarkable nonlinear optoplasmonic features can be developed in its wake. In this regard, it has been shown that nanostructures based on unit cells abiding to octupolar tensor symmetry such as inspired by trigonal and tetrahedral molecules allow for highly sensitive plasmonic devices.^36,51^ In the context of nonlinear optics, the term octupolar is related to the 3-fold symmetry and its implication on the quadratic nonlinear susceptibility tensor that is responsible for second harmonic generation. Octupolar molecules can exhibit very interesting optical properties, especially in their interaction with polarized light. Moreover, their rounded-off shape facilitates their packing in a periodic and compact lattice, as opposed to less favorable elongated rodlike dipolar molecules.^52,53^ In this work, we designed, simulated, and fabricated a 2D plasmonic nanostructure based on a novel octupolar pattern. The geometry proposed here results from a periodic arrangement of an octupolar unit cell (Figure 1A) made of three triangular nanocavities (NCs), with a side *L* of 180 nm and with their centers placed at vertices of a virtual triangle with side *S* of 230 nm, realized within a thin gold film. The lateral dimension of a cell is about 430 nm with a minimum intercell distance *D* of 100 nm. The geometry represents a hybrid metal–polymer multilayered system as shown in Figure 1B.

**Figure 2:**
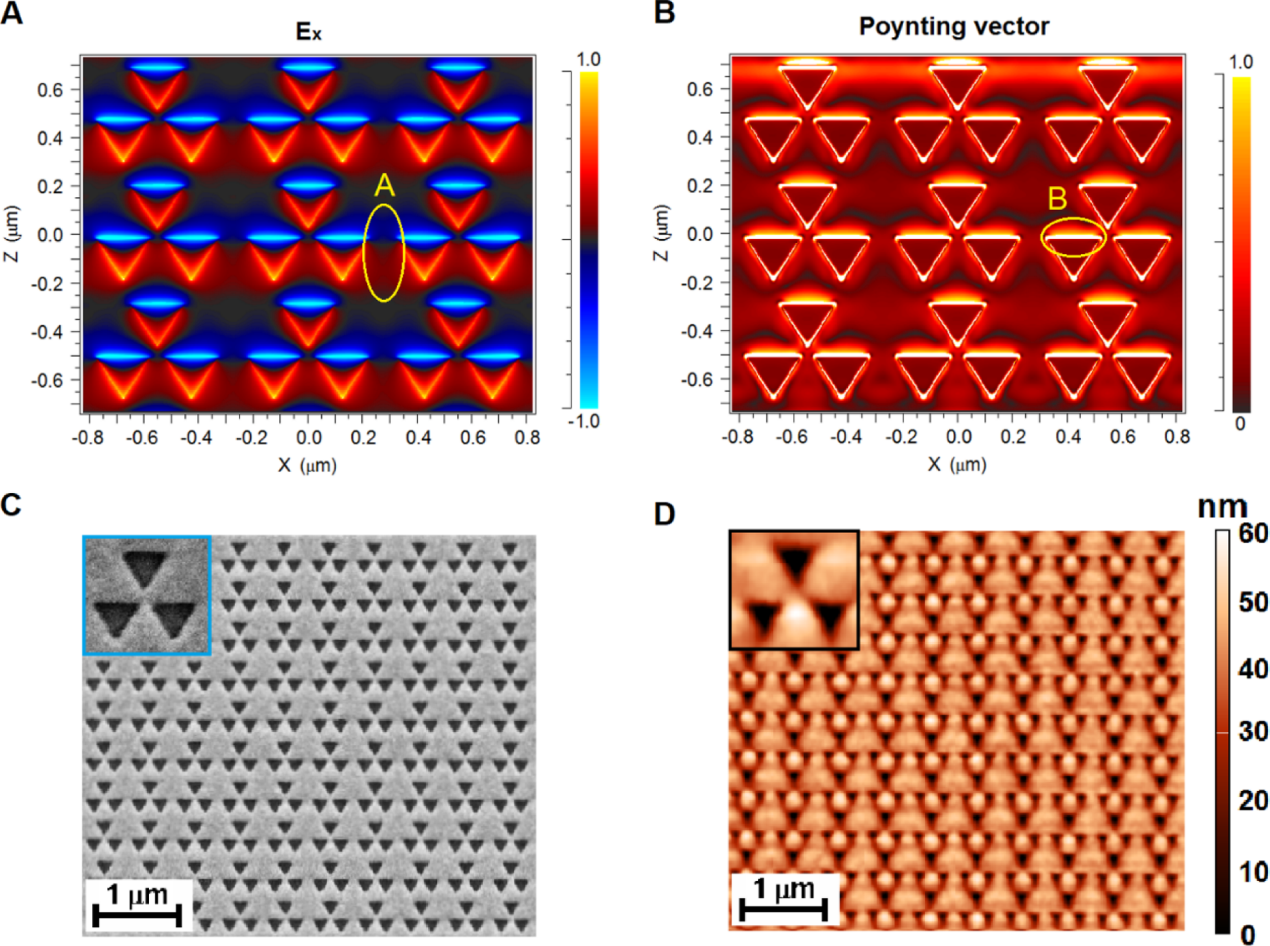
Analysis of the octupolar nanopattern taken into account. FDTD numerical simulations: (A) distribution of the EM x-component and (B) distribution of the Poynting vector intensity. Morphological analysis: (C) SEM image and (D) AFM image.

Figure 2 shows the near-field distributions calculated by the FDTD method applied to an octupolar nanopattern of interest with the EM x-component in (2A) and the Poynting vector intensity in (2B). A morphological analysis is displayed in (2C,D), respectively, from scanning electron microscopy and atomic force microscopy (AFM) imaging. In the simulations, the EM values are normalized with respect to their maxima. The field distribution is characterized by both a strong interaction between the neighboring cells (i.e., A region in yellow, Figure 2A) and regions with high intensity (hot spot) localized near the edges of the triangles (i.e., B region in yellow, Figure 2B).

Although these features cannot be directly linked to the detection properties, they represent important prerogatives that enable a plasmonic nanopattern for sensing applications. In particular, the EM interaction between cells enables a Pointing vector distribution more uniform and higher in average than, for example, in geometries based on a conventional unit cell comprising a single triangular element.^19^ From a physical point of view, the field properties of our multilayer are due to two types of surface plasmon resonance (SPR) modes: propagating SPP and LSPR present, respectively, on the top (plane A in Figure 1B) and on the lower (plane B in Figure 1B) layers of our device. Hence, the calculated field distribution is the superposition of two different effects generated by these modes: a long-range photonic coupling and a short-range dipole coupling. The near-field distribution in regions between the NC cells (i.e., A region in Figure 2A) is due mainly to the long-range coupling associated with the photonic diffraction of the SPP modes with the unit cells. Such diffraction produces an in-plane multi-scattering interaction that allows to increase the intensity of the field in these regions of the pattern. Otherwise, the near-field distribution in proximity of the NC edges (i.e., B region in Figure 2B) can be attributed to the short-range dipolar coupling associated with the LSPR modes generated between the neighboring NCs.

These peculiar properties of the field allow for higher photon–nanostructure interaction times (also known as dwelling times), increasing the probability of detecting an analyte and thus making these types of pattern promising for developing highly sensitive devices.

The analyzed nanopattern was fabricated using the EBL process that is known to guarantee the realization of structures with high resolution, accuracy, and repeatability. The fabrication process is mainly made of the usual lithographic steps used for nanostructures based on gold nanopillars but omitting the final lift-off procedure. As result of this simplified procedure, we achieved a hybrid multilayer (Figure 1B) with gold present also inside the NCs.

Differently from the case of Rippa et al., 2020,^52^ where an octupolar geometry with a lower gold filling factor was used to optimize a plasmonic signal measured in transmission, here, in the case of SERS measurements performed in reflection, the higher gold filling factor of the nanopattern allows three main advantages for sensing: an higher signal-noise ratio, shielding the fluorescence from the glass, and, more importantly, offering a greater gold surface to the linkage of molecules toward functionalization, thus enhancing the sensing performances. Morphological analysis shows that both the size of the triangular NCs and the minimum intercell distance are regular and well shaped on the whole pattern. From the AFM image, we can estimate an average depth for NCs of 55 ± 2 nm.

**Figure 3:**
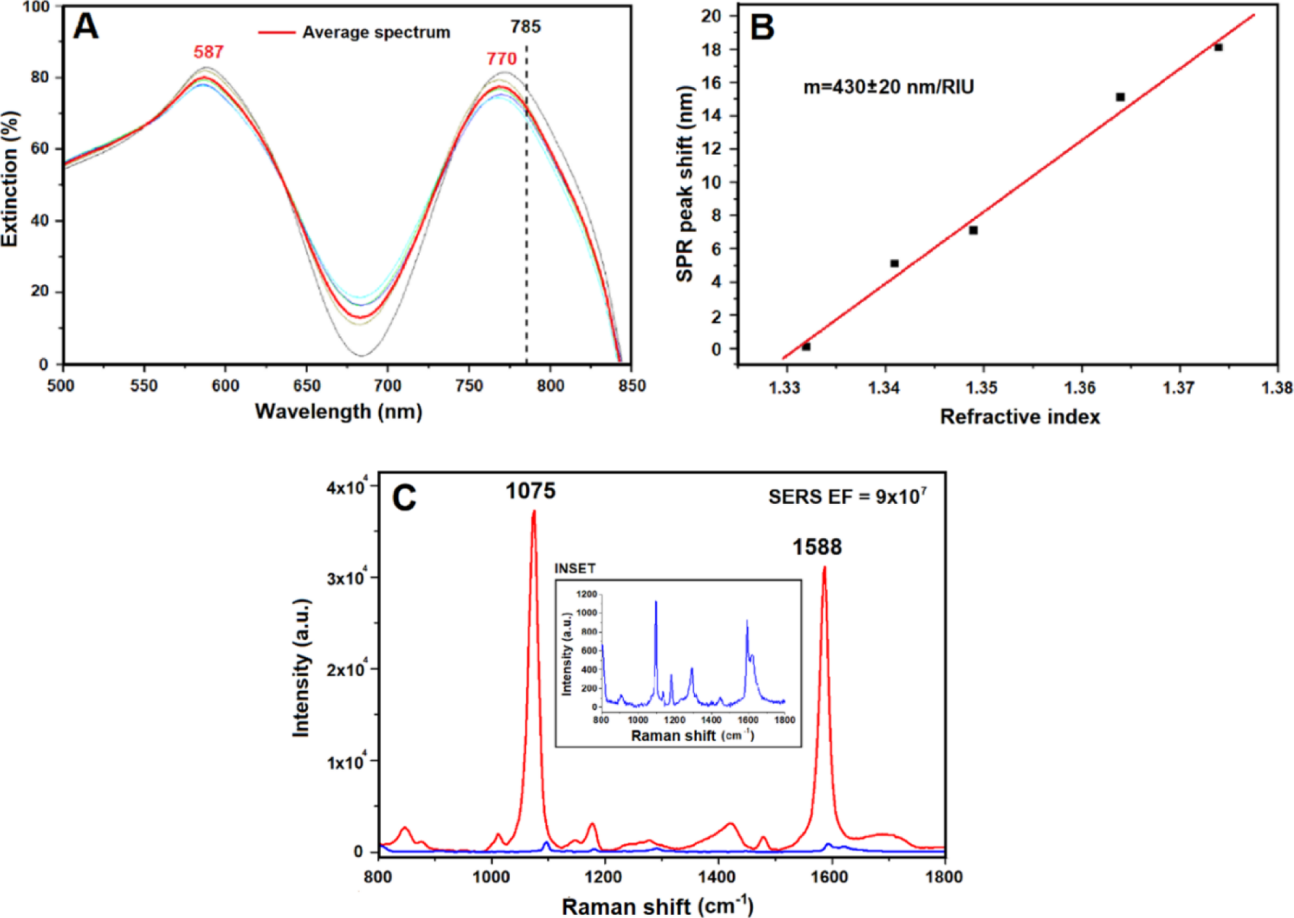
Spectroscopic characterization of the hybrid nanostructure based on octupolar geometry: (A) average extinction spectra (red curve), (B) SPR peak vs refractive indices of different medium, linear fit (red line), and (C) SERS spectrum achieved for a SAM of 4-MbA molecule (red line) and the Raman spectra of 4-MbA in the bulk (blue curve), further magnified in the inset.

Subsequent spectroscopic characterization of the obtained plasmon nanostructure allows us to test its performance for sensing applications. Two transduction principles were investigated: the vis–NIR extinction spectrum of the LSPR that depends on the analyte concentration and SERS that carries information not only about the analyte concentration but also about the interaction between the analyte and the substrate. The combination of both interrogation mechanisms in the same sample may lead to gains in selectivity as well as in specificity. The red curve in Figure 3A is the average extinction spectrum obtained from measurements on six replicas of the nanostructure under consideration.

The spectrum shows a double-resonance band with SPR peaks close to 587 nm (visible region) and 770 nm (infrared region). The double resonance shown by our pattern is mainly due to its structural nature made of gold nanocavities realized inside a thin gold film that supports complex modes generated from the interaction of both localized (LSPR) and propagated (SPR) resonances as discussed above. However, as shown in the literature,^54^ pattern with fractal geometries (as in the case of the octupolar cells here considered) can generate multiple resonances which can be explained by energy hybridization of the modes. It is worthwhile to underline that, although nanostructures with multiple resonances have already been studied and discussed in other works,^54^ the presence of a double-resonance band represents an additional important property that enables the octupolar pattern considered for a multispectral SERS investigation to analyze both metal and inorganic oxides using a visible excitation source and biological analytes using an infrared excitation source. Figure 3B shows the experimental trend of the shift (Δλs) of the SPR peak found in the infrared region (λ_peak_ = 770 nm) versus the refractive index (*n*) of different media performed as described in the Methods section. We estimated the bulk sensitivity *m* through the linear fit Δλs* * = *m*Δ*n* (red line in Figure 3B) achieving a value of 430 ± 20 nm/RIU. This latter value represents one of the highest achieved in the literature in the case of resonance shifts measured in transmission without the use of prism for the coupling of light. Successively, we evaluated the SERS performance of the octupolar nanopattern estimating the EF defined as EF = (*I*_SERS_ × *N*_REF_)/(*I*_REF_ × *N*_SERS_) using a self-assembled reference monolayer (SAM) of the molecular probe 4-MbA at an excitation wavelength of 785 nm. In the EF formula, *I*_SERS_ and *I*_REF_ are the intensities of the peak at 1076 cm^–1^ in the SERS and Raman spectrum of 4-MbA, respectively, while *N*_SERS_ and *N*_REF_ are the number of molecules contributing to these signals. These last parameters have been estimated using the physical and chemical properties of 4-MbA and following the approach reported in detail in an earlier work^55^ (details are reported in the Supporting Information). Figure 3C shows both the SERS spectra for the 4-MbA SAM (red line) and the Raman spectra of the molecule in bulk powder (blue curve), both obtained from averaging repeated measurements at 10 different points of the samples. From these experimental evaluations, an average EF value for the nanostructure investigated of ∼9 × 10^7^ was achieved.

In the study of Rippa et al., 2017,^51^ an octupolar geometry based on an arrangement of air nanocavities inside a thin gold film in the hybrid multilayer configuration was used. However, even if this last pattern can be considered geometrically quite similar to that analyzed in the present work, here we have experimentally achieved an average SERS EF higher (almost double) than the maximum value of 5 × 10^7^ estimated in the case of the geometry taken into account in Rippa et al., 2017. This higher SERS EF can be explained by comparing the plasmonic resonances of the two nanostructures considered. In the case of the pattern in Rippa et al., 2017, the resonance peak measured in air was at a wavelength less than 750 nm, while in the case of the geometry considered in this work, the peak is around 770 nm. In this latter case, therefore, there is a better fit between the wavelength of the laser source at 785 nm used for the SERS measurements and the resonance peak itself, thus allowing a more efficient analysis characterized by higher signal amplification. Moreover, as an added benefit, the geometry considered in this work characterized by a unit cell made of three equal triangles, without the presence of an additional small central triangle as in the case of the nanopattern in Rippa et al., 2017, turns out to be easier and faster to be produced and replicated by the EBL technology.

### SERS Measurements for Stx2a Detection

**Figure 4:**
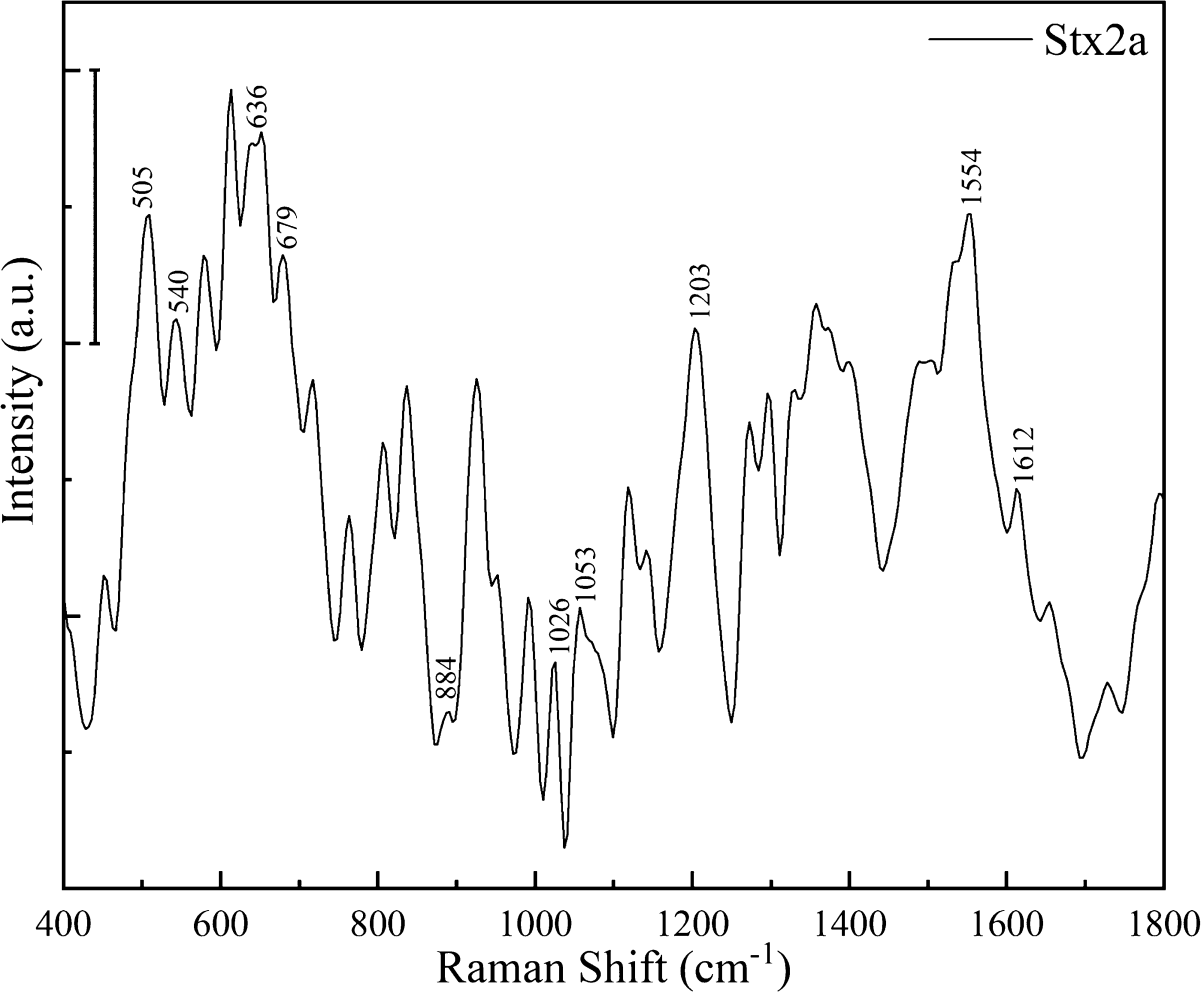
SERS measurements on the Shiga toxin. Spectra of Stx2a adsorbed on the octupolar nanostructure (154 nM). The scale bar represents 2000 a.u. The spectra were smoothed using an Origin-pro 2020 using the Savitzky-Golay algorithm with seven points of window.

As a case of study, the plasmonic nanostructure was tested as a SERS device to identify and characterize Stx2a, achieving a SERS fingerprint spectrum of the protein. The toxin was adsorbed directly on the nanostructure using the experimental parameters described in the Methods section, and reproducible spectra were found with resolved peaks characteristic of the toxin. In Figure 4, we show the fingerprint signal depicting Stx2a achieved by a mean of 10 different acquisitions.

It is worthwhile to underline that the reported spectrum (Figure 4) refers to a final assayed toxin concentration of 154 nM. Indeed, the nominal concentration of the Stx2a samples (550 nM), calculated by considering the dilution factor employed for the stock solution (see the subsection SERS Substrate with the Shiga Toxin: Functionalization Procedure in Methodssection), was corrected by considering the loss of toxin due to its nonspecific adsorption to the plastic surface. For this reason, the enzymatic activity of our water-diluted toxin samples was assayed^56^ and compared with that of a parallel toxin sample diluted in phosphate-buffered saline (PBS) added with 1% bovine serum albumin (BSA). This comparison allowed us to estimate a protein recovery of 28%, and this factor was considered to calculate the real toxin concentration of our samples.

The spectrum registered upon incubation of 154 nM Stx2a onto our metasurface (Figure 4) is 3 orders of magnitude smaller than the LOD for amino acids (100 mM) and 1 order smaller than the LOD for BSA (1 mM) achieved with Raman spectroscopy reported in the literature.^57−62^ The identified peaks were assigned referring to previously published articles (Table 1). The six disulfide bonds present in Stx2a (one in A subunit and one in each of the five B subunits) are clearly resolved in the presented spectra showing a major peak (505 cm^–1^, Figure 4) and a secondary minor peak with higher frequency (540 cm^–1^, Figure 4), thereby indicating the presence of different conformations of the S–S groups. The C–S stretching region is dominated by the C–S bond vibration of cysteine (679 cm^–1^) though the peak of methionine (717 cm^–1^) is resolved. As expected, from the spectra, it is possible to identify the peaks related to the aromatic amino acids Trp (552, 764, 883, 1053, and 1554 cm^–1^), Tyr (636, 837, 856, 1203, and 1612 cm^–1^), and Phe (1026, 1203, and 1612 cm^–1^). The typical Tyr doublet (837 and 856 cm^–1^) due to Fermi resonance shows a more intense line of lower frequency. The amide III vibration corresponding to the α-helix conformation (1260–1300 cm^–1^), well represented (1269 and 1300 cm^–1^) in the spectra, is in accordance to previous crystallographic studies and circular dichroism analysis, showing that Stx2a contains a significant fraction of the α-helix structure. The shoulder band relative to the amide I mode (1658 cm^–1^) confirms this conclusion. We also want to point out that the toxin can be oriented in different ways on the nanostructure, and the final spectrum is an average linked to such different orientations, which correspond to repeatable measurements providing a complex spectrum.

**Table 1:** Tentative Assignation to the Bands of Stx2a Spectra and Shared Peaks between the Toxin Alone and the Ab + Stx2a Complex

wavenumber cm^–1^	assignation Stx2a	shared with Ab + Stx2a
**505–540**	protein S–S^57−59^	**500–550**
552	Trp	**552**
**579**	Nd	579
**613**	ring breathing^63^	613
**636**	Tyr ring deformation^57^	**640**
**652**	ring breathing^63^	656
**679**	C–S cysteine^64^	
**717**	met C–S stretching^64^	
**764**	Trp ring breathing^60^	**768**
**810**	nd	
**837**	Tyr	
856	ring Tyr^57^	**856**
**883**	PBS or Trp^60^	
**918**	CC stretching^61^	**995**
**995**	amide III^65^	**1022**
**1026**	in-plane ring CH def. Phe^62^	
**1053**	Trp or Glu^66^	**1053**
**1115**	stretching (CN) protein^60^	1115
**1203**	Phe,^64^ Tyr^60^	**1200**
**1269**	amide III alpha helix^60,67,68^	
**1300**	amide III alpha helix^60,67,68^	**1300**
**1354**	wagging (CH2,CH3)^65^	1358
1392	aromatic amino acids COO^–^ stretching^57^	**1392**
**1554**	Trp^62^	
**1612**	bending C=C in Phe and Tyr^57,58,64^	1616
**1658**	amide I,^60,62^ alpha helix^64^	1655

**Figure 5:**
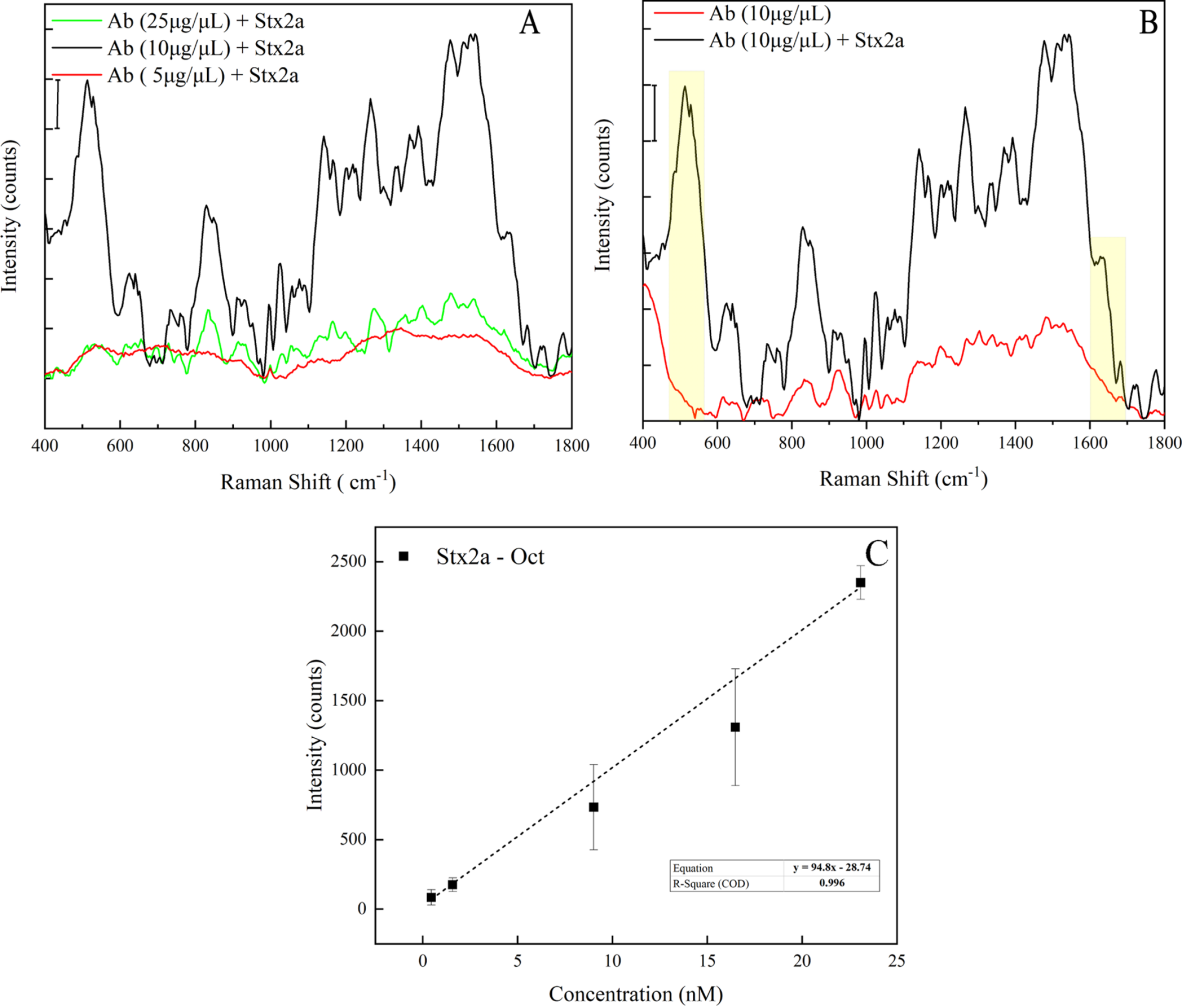
SERS measurements of the Shiga toxin performed with the immunosensor. (A) Comparison between the spectra of the toxin captured by the antibody (Ab + Stx2a) achieved with three different antibody (Ab) concentrations: 5, 10, and 25 μg/mL, the scale bar represents 2000 a.u. (B) Comparison between the spectra of the Ab + Stx2a complex and the antibody alone. Two spectral bands (460–590 and 1616–1660 cm^–1^) highlighted in yellow correlated with the interaction between the antibody and toxin. (C) Calibration curve achieved by plotting the 1554 cm^–1^ peak intensity vs the toxin concentrations.

In order to develop an immunosensor for a specific Stx2A detection, an anti-Stx2a antibody (Ab) was used to functionalize our SERS substrate. To optimize the capture, three antibody concentrations 5, 10, and 25 μg/mL were tested and incubated on three equal replicas of the nanostructured substrate. After a washing step, the toxin was applied on the functionalized substrates and allowed to react with the immunosurface for 1 h. The samples were subsequently washed, dried, and finally tested. We found that the highest signal amplification was obtained with 10 μg/mL of Ab absorbed on the sensor (Figure 5A).

For this concentration, the spectral range between 460 and 590 cm^–1^, taken as a reference, was 7.5-fold higher than the samples at 5 or 25 μg/mL. Interestingly, the comparison between the spectra of the Ab alone and Ab + Stx2a shows that two main spectral bands 460–590 and 1616–1660 cm^–1^ appear to be correlated with the antibody–toxin interaction (yellow bands in Figure 5B). It is possible that in the toxin/antibody complex, the disulfide bonds become more viable due to the conformational changes. The comparison between the spectrum achieved with Stx2a adsorbed onto the naked gold metasurface and that of Stx2a captured by the immunosurface (Figure S1, Supporting Information) shows some common peaks, probably identifying the features of the toxin as displayed in Table 1.

Subsequently, the sensing performance of our immunosystem was tested with different toxin dilutions (1, 5, 16.5, 33, and 55 nM) by recording the SERS spectra achieved for the different true toxin concentrations (0.44, 1.57, 9.01, 16.47, and 23.09) determined by directly assessing the enzymatic activity of Stx2a as described above.^56,69^ Higher concentration of the analyte resulted in a higher SERS signal amplification. The enhancement involved the whole spectrum, and the amplification of the band centered at 1554 cm^–1^ (associated with Trp, see Table 1) was chosen to evaluate the LOD of the immunosurface. For this purpose, all the SERS spectra were referenced to the zero level. The 1554 cm^–1^ peak intensity versus the toxin concentrations was plotted to obtain a calibration curve (Figure 5C). The LOD calculated from a residual standard deviation of the regression line was 1.4 nM.

The assay performance of the developed immune system was compared with the gold standard ELISA^70^ targeting the toxin in the same dilution range (Figure S2A, Supporting Information). Since ELISA-based signals are linear over a narrow range of lower toxin concentrations (up to 0.15 nM), 70 small aliquots (0.75 μL) of the water-diluted Stx2a samples were added to the ELISA plate and brought to the volume required for the assay (100 μL) with PBS–BSA 1%. The responses of the ELISA-based and SERS-based data points were comparable, showing that the calibration curves generated were linear (*R*^2^ > 0.99) with similar slopes (Figure S2A). A regression graph was used to compare the two analytical methods (Figure S2B). Each point of the graph represents the analytical response of ELISA (*x*-axis) and SERS (*y*-axis), weighted over the standard deviation, to a given concentration. The *R*^2^ = 1 indicates that the responses of the two methods are linear. The slope slightly higher than 1 (*b* = 1.2) indicates that the immunosensor and ELISA results are proportional, although a slight overestimation occurs with our method (Figure S2B). Finally, the specificity of the proposed immunosystem was tested by considering a nontarget protein, human/BSA (H/BSA). The SERS spectra recorded upon 4.5 μM H/BSA incubation (about an order of magnitude higher than the greatest toxin concentration measured) are practically identical to that of the anti-toxin antibody (Figure S3, Supporting Information). This result demonstrates the lack of H/BSA capture by the antibody and represents a significant proof of the specificity of our immunosurface.

## Conclusions

In conclusion, this work explores the plasmonic properties of a novel doubly resonant octupolar geometry showing both numerically and experimentally the potential of nanostructures based on this design to realize advanced devices. Through FDTD simulations, we discussed the physical properties characterizing the near-field distribution resulting from this geometry and reported the high experimental values of important figures of merit such as SERS EF and SPR bulk sensitivity and the presence of a peculiar double resonance in the visible and infrared region. These properties make it possible to use the octupolar geometry investigated to engineer high-performance detection devices including a dual-mode configuration for a SERS-SPR sensing chip. As a benchmarking case, we demonstrated the efficacy of our nanopattern to realize both the SERS analysis of the Stx2a fingerprint and the SERS immunosensing for the specific toxin detection recorded at low nM concentrations.

## Methods

### FDTD-Based Simulations

We investigated the near-field distribution of the octupolar geometry considered performing a three-dimensional numerical simulation using the FDTD method. We used the commercial software R-Soft and its calculation tool FullWAVE (Design group Inc). During the calculation, we reproduce the multilayer shown in Figure 1B with a semi-infinite air cover, a semi-infinite glass substrate (BK7), and a thickness of 50, 2, 180, and 15 nm for gold, chrome, ZEP, and ITO, respectively. An incident Gaussian source with a wavelength of 785 nm, polarized in the plane of the NCs and that propagates perpendicular to it, was used to excite the patterns. Real refractive indices were considered for air (*n*_air_ = 1), glass (*n*_bk7_ = 1.51), ZEP (*n*_ZEP_ = 1.55), and ITO (*n*_ITO_ = 1.62), whereas complex indices were considered for chrome (*n*_CH_ = 3.13 and *k*_CH_ = 3.44) and gold (*n*_AU_ = 0.15 and *k*_AU_ = 4.78). Periodic boundary conditions in the plane of the NCs were used to simulate an infinite lattice based on the repetition of the unit cell, whereas the perfectly matched layer (PML) condition was considered in the direction perpendicular to the layers. The numerical results converge using a uniform spatial grid with a step size of 3 nm in each direction and a time step of 1.7 × 10^–3^ μm (in units of ct). Both electromagnetic x-component and Poynting vector distributions were calculated at the NC surface (plane A in Figure 1B) and are shown in Figure 2.

### Octupolar Nanostructure Fabrication

EBL (Raith 150) was used to nanopattern a 180 nm-thick layer of electron-sensitive polymer resist, styrene methyl acrylate (ZEP 520A), previously spin-coated on a 15 nm-thick ITO-coated glass substrate. The polymer layer was exposed to a 26 pA electron beam current and backed at 170 °C for 5 min. The octupolar pattern made of NCs was achieved in the ZEP layer after developing it in an n-amyl acetate solvent and rinsing for 90s in a 1:3 MIBK/IPA solution (methyl isobutyl ketone/isopropyl alcohol), then washed by IPA solution, and dried in a gentle flow of argon gas. Two nanometer Cr and 50 nm Au films were evaporated successively on the resist surface by the use of the SISTEC CL-400C e-beam system. Using this procedure, we fabricated different replicas of nanostructures based on the octupolar geometry taken into account. The size of the nanopatterned area was 300 μm × 300 μm. The geometry of the pattern consists of a periodic array of a main unit cell (Figure 1A) made of three triangular NCs with side *L* = 200 nm and with their centers placed at vertices of a virtual triangle with side *S* = 230 nm. The minimum intercell distance was *D* = 100 nm.

### Spectroscopic Characterization

Spectroscopic characterization of the octupolar nanostructure considered was performed by vis–NIR extinction measurements. We used the setup reported and described in previous works.^17^ We evaluated the extinction spectra Es versus the wavelength λ achieved from the nanostructure when unpolarized light of a halogen source is focused on it by an objective (10×, N.A. = 0.25). First, we measured the transmission signal *T*(λ) by means of a fiber with a core of 50 μm connected to a spectrophotometer (Ocean Optics USB4000, optical resolution ∼1 nm). The percentage transmission *T* % (λ) was calculated with the relation *T* % (λ) = (*T*(λ) – *T*_d_)/(*T*_ref_(λ) – *T*_d_) × 100, where *T*_ref_(λ) represents the reference spectrum recorded through the multilayer but out of the nanostructure and *T*_d_ represents the dark spectrum obtained by switching off the light source. Finally, Es was achieved by the expression Es(λ) = 100 −*T* % (λ), and from it, the SPR peak was determined. We evaluated the bulk refractive index sensitivity of the nanostructure by measuring the shift of the SPR peak found in the infrared region (λ_peak_ = 770 nm) for five different media: water (*n* = 1.332), IPA/water 1:10 wt/wt (*n* = 1.341), IPA/water 1:5 wt/wt (*n* = 1.349), IPA/water 1:1 wt/wt (*n* = 1.364), and IPA (*n* = 1.374). The refractive indices of the media were measured using an Abbe refractometer.

### SERS Measurements

SERS analysis was performed using a Raman system (QE Pro-Raman, Ocean Optics) coupled with an upright microscope (Olympus BX51) in a backscattering configuration. The Raman system was configured for λ = 785 nm with a grating of 1200 lines/mm and an input slit of 50 μm. The spectra were collected in the range of 400–2000 cm^–1^ using an acquisition time of 10 s, a 50× (N.A. = 0.75) objective, and a laser power in the range of 10–36 mW.

### Toxin Extraction and Analysis

*E. coli* C600 (933 W) producing the Shiga toxin 2a (Stx2a) was a generous gift from Alison O’Brien (Bethesda, MD, USA). Stx2a was isolated by receptor analogue affinity chromatography on a Galα1-4Galβ-O-spacer–BSA–Sepharose 4B (Glycorex, Lund, Sweden) as previously described.^56^ After purification, the toxin preparation was passed through an ActiCleanEtox column (Sterogene Bioseparations, Carlsbad, CA, USA) to remove the trace endotoxin contaminants. Purified Stx2a was analyzed by sodium dodecyl sulfate polyacrylamide gel electrophoresis in reducing conditions and stored in PBS at −80 °C in aliquots. Mouse anti-Stx2a monoclonal Ab (Stx2-BB12) was purchased from Toxin Technology (Sarasota, FL, USA).

### SERS Substrate with the Shiga Toxin: Functionalization Procedure

SERS measurements of Stx2a were performed using two different strategies: (a) the direct deposition of the toxin on the nanostructure and (b) pre-functionalizing the gold surface of the substrate with the anti-Stx2a Ab monoclonal antibody to achieve a specific capture of the toxin. For the preparations reported below, we used stock solutions of 6.62 μM Stx2a toxin in ddH_2_O and 1 mg/mL of anti-Stx2a Ab toxin monoclonal antibody reconstructed in ddH_2_O stored at −20 °C until use. Stx2a in ddH_2_0 was obtained by repeated concentration/dilution cycles by centrifuging on Centricon 30 filters (0.5 mL) for 3 min at 12,000g. For the direct deposition of Stx2a, the toxin stock solution (1 μL) was diluted in ddH_2_O down to the theoretical concentration of 550 nM. Then, 3 μL of the toxin dilution was dropped on the nanostructure corresponding to ∼100 ng (∼10^12^ molecules) of Stx2a (assumed molecular mass = 68,000 Da). The sample was left to dry at room temperature before SERS measurements. Since diluting Stx2a in the absence of a protein carrier induces loss of toxin due to nonspecific binding to plastic surfaces, the enzymatic activity of the water-diluted toxin was assayed^56^ and compared with that of a parallel toxin sample diluted in PBS containing 1% BSA. The calculated recovery was 28% corresponding to a true final toxin concentration of 154 nM, that is, ∼30 ng (∼2.8 × 10^11^ molecules) of Stx2a on the nanostructure. For the specific capture of the toxin, the immunofunctionalization of the substrate was achieved through the use of a physicochemical adsorption approach. We tested three different concentrations of anti-Stx2a Ab: 5, 10, and 25 μg/mL. The antibody solutions were prepared by dilution of the stock with PBS (pH 7.4) and dropped (3 μL) on three different replicas of the octupolar nanostructure realized on the same glass substrate. After overnight incubation in a humid chamber at room temperature, the sample was washed five times with 1 mL of PBS (pH 7.4) and dried with N_2_ before SERS analysis. Successively, the functionalized nanostructures were tested to detect different theoretical concentrations of Stx2a (1–550 nM) dropped (3 μL) on the substrate. The true final concentration of each dilution was determined by assessing the enzymatic activity of Stx2a^56,69^ (see above) and reported in the Results and Discussion sections. Before SERS measurements, the sample was left for 1 h under incubation in a humid chamber at room temperature and washed five times with 1 mL of PBS (pH 7.4) and dried with N_2_. The biosensor specificity was tested using BSA proteins −BSA– (Biowest P6154). Specifically, 4.5 μm of BSA in PBS pH 7.4 was deposited on the biosensor surface for 1 h in a humid chamber at room temperature. After the incubation, the biosensor was washed five times with 1 mL of PBS pH 7.4 and dried with N_2_ before SERS spectrum registration.

Water-diluted Stx2a was also assayed by ELISA^70^ according to the manufacturer’s instructions (cat. no. 542010 Eurofins Abraxis, Warminster, PA, USA)..

## Supporting Information Available

Comparison between the spectra of Stx2a alone and the Ab + Stx2a complex (154 nM); comparison between the regression lines obtained by detecting toxin samples with Elisa and SERS sensor; comparison of methods’ performances via a scatter diagram; and specificity evaluation (PDF)
