# Supplementary material for: Plasmonic Metasurfaces for Specific SERS Detection of Shiga Toxins
Source: ACS Appl Mater Interfaces. 2022 Jan 19;14(4):4969–79. doi: 10.1021/acsami.1c21553 (PMC8815041; doi:10.1021/acsami.1c21553)
Supplement: Supplementary file 1 — am1c21553_si_001.pdf [file am1c21553_si_001.pdf]

Supporting Information: Plasmonic metasurfaces for specific SERS detection of Shiga Toxin

M. Rippa[1⊥], D. Sagnelli[1⊥], A. Vestri[1], V. Marchesano[1], B. Munari[2], D. Carnicelli[2], E. Varrone[2], M. Brigotti[2,1*], R. Tozzoli[3], M. Montalbano[3], S. Morabito[3,1], J. Zhou[4], J. Zyss[5,1] and L. Petti[1*]

[1]Institute of Applied Sciences and Intelligent Systems "E. Caianiello" CNR, Pozzuoli, Italy

[2]Dipartimento di Medicina Specialistica, Diagnostica e Sperimentale, Sede di Patologia Generale, Università di Bologna, Bologna, Italy

[3]Department of Food Safety, Nutrition and Veterinary Public Health, Istituto Superiore di Sanitá, Rome, Italy

[4]Institute of Photonics, Faculty of Science, Ningbo University, Ningbo, People's Republic of China

[5] Lumière, Matière et Interfaces (LUMIN) Laboratory, Institut d'Alembert, Ecole Normale Supérieure Paris-Saclay, Université Paris Saclay, Gif sur Yvette, France

E-mail: l.petti@isasi.cnr.it; maurizio.brigotti@unibo.it

- **EF calculation and SERS measurements**

The Raman enhancement factor (EF) of the SERS-active substrates was determined using the following equation:

$E_F = (I_s \times N_r)/(I_r \times N_s)$, (1)

where I_s and I_r are respectively the integrated intensities of the main SERS peak at 1073 cm^{-1} of 4MBA molecules adsorbed on the different substrates and the area of the peak at 1073 cm^{-1} of the bulk in the Raman spectrum, while N_s and N_r are the number of 4MBA molecules contributing to the signal in the two cases considered at the irradiation spot of the laser. I_r was calculated to be 2.72 x 10^3. The values of I_s were calculated to 8.48 x 10^5 for the nanostructures (SERS spectra of Figure 3C). The number of molecules that contribute to the SERS signals is dependent on the area of the laser spot (A_{laser}), on the filling factor (FF) and on the cross section for 4-MBA (σ), as indicated in the following equation 2 [60]:

$N_s = A_{laser} * FF/\sigma$ (2)

So that the $N_s = 0{,}76 \times 10^6$. The number of molecules that contribute to the Raman bulk signal is given by the following equation 3 [60]:

$N_r = V_{exc} * N_{Av} * D_{4MBA} / W_{4MBA}$ (3)

with the excitation volume $V_{exc} = 38.4$ μm^3, the Avogadro's number is $N_{av} = 6.022 \times 10^{23}$ mol^{-1}, the density of 4-MBA is $D_{4MBA} = 1.5$ g cm^{-3}, the molecular weight for 4-MBA is $W_{4MBA} = 154{,}19$ g mol^{-1}. So that, $N_r = 2.2 \times 10^{11}$.

Finally, the calculated value for the SERS enhancement is obtained to be 9×10^7.

- **SERS spectra of the Stx2a on the naked metasurface and on the immuno-surface**

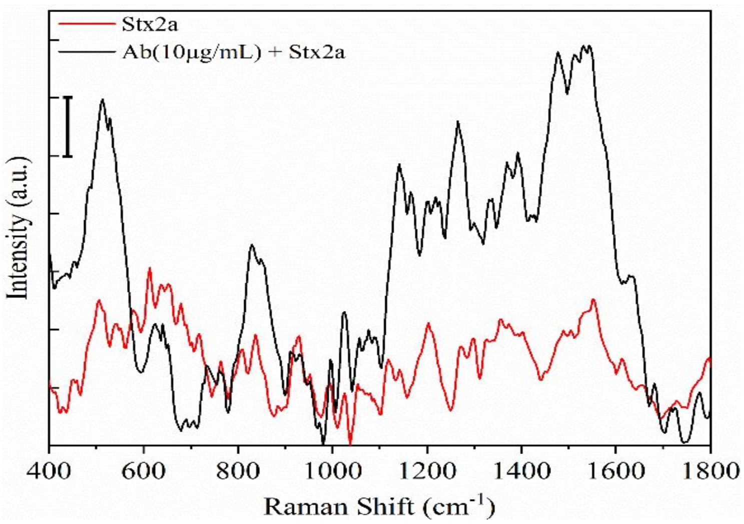

Figure S1. Comparison between the spectra of the Stx2a alone and Ab+Stx2a complex (154 nM).

- **Immuno sensor and gold standard comparison**

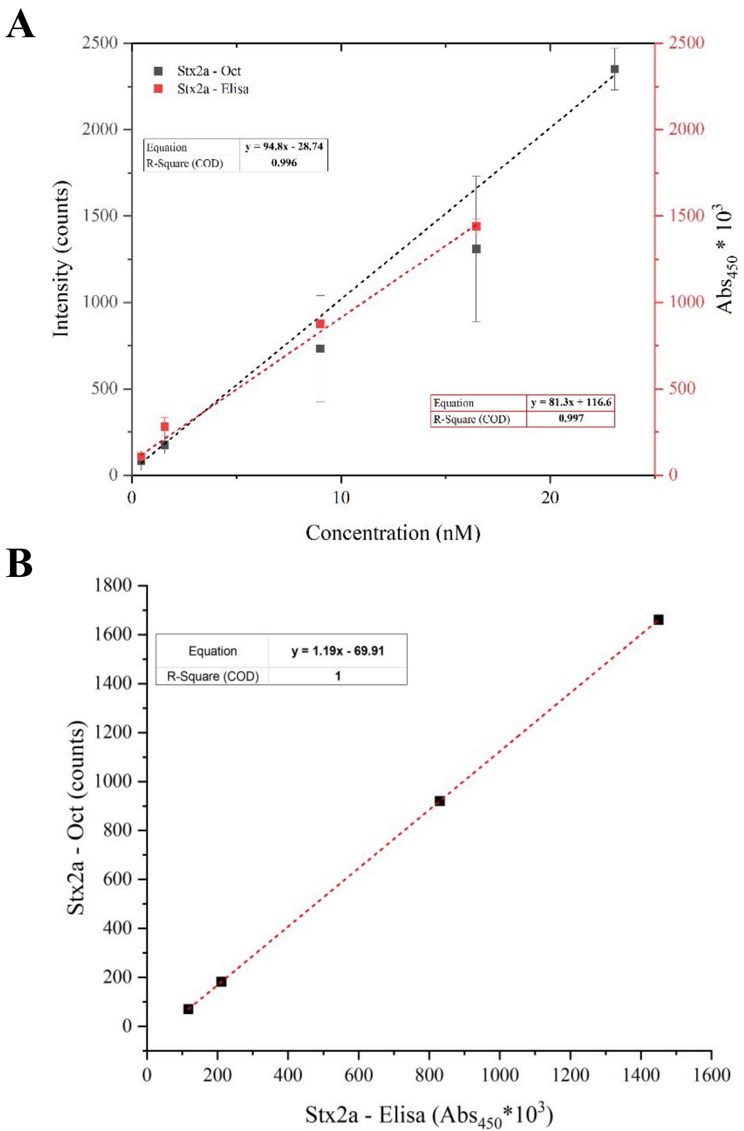

Figure S2. A: Comparison between the regression lines obtained by detecting toxin samples with Elisa and SERS sensor. The Stx2a samples (theoretical concentration 1-55 nM) were assayed by the immune-sensor and by ELISA. The true toxin concentrations (0.44 -23.1 nM) were plotted against the ELISA signals (absorbance at 450 nm) and by SERS (counts). To compare the curves at the same order of magnitude, the absorbance values were multiplied by 10^3. B: Comparison of methods performances via a scatter diagram, plotting the weighted responses across the calibration concentrations. This graph shows that the methods are linear (R^2=1) and proportional to each other with a slight overestimation by the immune-sensor (m>1).

- **Specificity measurements**

We evaluated the specificity of the proposed immuno-system by testing a non-target protein, i.e. the bovine serum albumin (BSA). The spectrum recorded upon BSA (4.5µM, about an order of magnitude higher than the greatest toxin concentration tested) incubation is practically identical to the anti-toxin antibody spectrum, as shown in the figure S3.

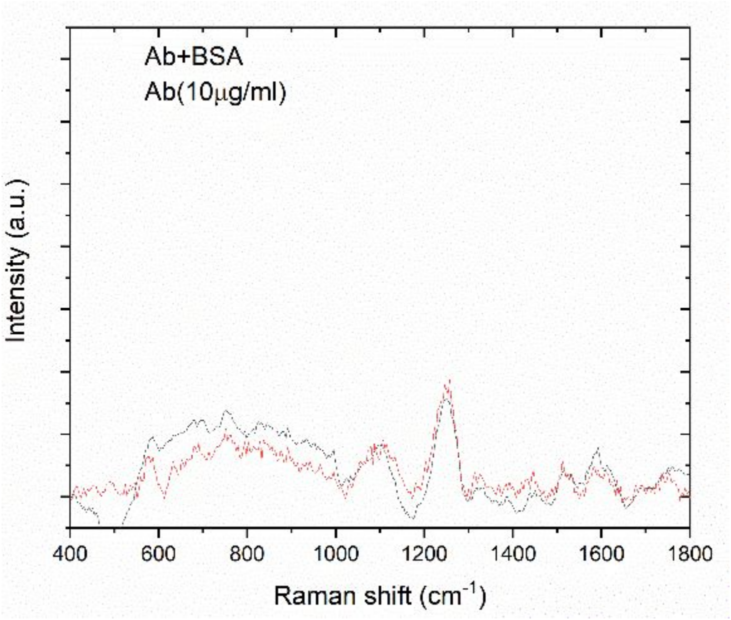

Figure S3: Specificity evaluation: in red the SERS spectra of the antibody (Ab), in black the SERS spectra achieved with Ab+BSA.
